# Influence of Spray-Drying and Room Temperature Storage on the Anti- and Prooxidant Properties of Fermented Juçara Pulp

**DOI:** 10.17113/ftb.58.01.20.6335

**Published:** 2020-03

**Authors:** Karla Bigetti Guergoletto, Kamila Landucci Bonifácio, Décio Sabbatini Barbosa, Daniel Farinha Valezi, Aroldo Salviato, Eduardo Di Mauro, Elza Iouko Ida, Sandra Garcia

**Affiliations:** 1 Department of Food Science and Technology State University of Londrina, Celso Garcia Cid, Road – Pr 445 Km 380, Londrina, Paraná, Brazil; 2 Department of Pathology, Clinical and Toxicological Analysis State University of Londrina, Celso Garcia Cid Road – Pr 445 Km 380, Londrina, Paraná, Brazil; 3 Physics Department; State University of Londrina, Celso Garcia Cid Road – Pr 445 Km 380, Londrina, Paraná, Brazil

**Keywords:** *Euterpe edulis* Mart., *Lactobacillus reuteri*, *Lactobacillus plantarum*, fermented food, dehydrated fruit

## Abstract

Many fruits and vegetables contain compounds with antioxidant properties, but the processing and storage conditions of the food industry may damage these beneficial compounds and produce free radicals that are associated with oxidative stress. This study aims to evaluate *in vitro* the antioxidant capacity and prooxidant effects of juçara pulp fermented with *Lactobacillus reuteri* or *Lactobacillus plantarum* before and after spray-drying with maltodextrin, gum arabic or gelatin and stored at 25 °C for 90 days. The antioxidant capacity was assessed by measuring the ability to scavenge reactive oxygen species (ROS) in the neutrophil respiratory burst and free radical 2,2-diphenyl-1-picryl-hydrazyl (DPPH), and by determining the total phenolic content. The prooxidant effects were analyzed as free radical formation measured by electronic paramagnetic resonance (EPR). Fermentation by both bacteria increased the antioxidant activity, while the spray-drying process decreased the content of phenolic compounds (65-85%) and the DPPH scavenging ability, depending on the carrier usage. All of the samples inhibited ROS in the neutrophil burst, and the juçara pulp fermented by *L*. *reuteri* and dried with gum arabic exhibited the best performance. Spray-drying did not influence the intensity or type of free radicals detected by EPR. However, storage at room temperature decreased the antioxidant capacity and increased free radical formation.

## INTRODUCTION

The etiology of many chronic diseases (*e.g*. Parkinson’s, Alzheimer’s, and cancer) is associated with an imbalance in the cellular redox system and increased levels of reactive oxygen species (ROS) and reactive nitrogen species (RNS) (*1*). An increase in these species and a decrease in antioxidant defense mechanisms triggers oxidative stress, which causes damage and dysfunction in the components necessary for cellular maintenance (*2*).

To estimate the antioxidant capacity of food and biological fluids, more than one technique is often required because the methods are limited and differ in their duration and quantification (*3*). Generally, indirect methods are used more frequently than direct ones and among the usual is the DPPH test, based on the capability of stable free radical 2,2-diphenyl-1-picrylhydrazyl to react with H-donors. Folin–Ciocalteu assay is one of the oldest methods designed to determine the total content of phenolics, based on absorbance readings at *λ*=750 nm and frequently expressed in gallic acid equivalents (*3*). The method of reactive oxygen species production by the neutrophils is based on the fact that neutrophils are able to destroy invading cells through the production of Eros (essential for reactive oxygen species) protein, increasing the consumption of oxygen during phagocytosis, called respiratory burst. Oxygen is then used by a series of enzymes, leading to the production of free radicals and to oxidative stress. When neutrophils are stimulated, the produced Eros can oxidize the reagent luminol by transferring these chemical species to a state of electronic excitation, which results in the emission of light (*4*).

Many fruits and vegetables contain compounds with antioxidant properties, such as phenolic compounds, carotenoids, anthocyanins and tocopherols, which act as reducing agents, metal chelators, singlet oxygen suppressors, or hydrogen donors, inhibiting or reducing the stress-inducing reactions in cells (*5*). The pulp of the açaí fruit (*Euterpe oleracea* Mart. and *Euterpe precatoria* Mart.) has drawn great interest for its antioxidant compounds. Juçara (*Euterpe edulis* Mart.) is a palm tree in the açaí family that grows in the Atlantic Forest region, Brazil, and it has recently emerged as a promising source of natural antioxidants (*6*, *7*). The pulp of juçara and açaí is highly perishable, so processing is essential to increase its shelf life. However, industrial food processing may influence its antioxidant potential, as some compounds (*i.e.* anthocyanins) are unstable during processing and storage (*8*).

Spray-drying is a widely used food processing technique that transforms liquid food into powder using brief exposures to high temperatures. The products obtained with this technique are of high quality, have low water activity, and are easily handled, transported and stored (*9*). However, the high temperatures may change the composition of the food and induce oxidation of the naturally occurring compounds, leading to free radical formation that can trigger oxidative reactions and degradation (*10*). The influence of temperature on the antioxidant properties of plant products is complex, depending not only on the peak temperature but also on the time of exposure (*10*). Many studies have shown that drying agents can preserve the antioxidant capacity and maintain the bioactive compounds during high-temperature spray-drying, and may even create new antioxidant compounds (*8*, *11*, *12*).

Fermentation is a common food-processing technique, and many products use *Lactobacillus* spp., which have been associated with numerous health benefits (*13*). Foods fermented by these bacteria also increase their antioxidant capacity, making them more attractive to consumers (*14*, *15*). In a previous study, Guergoletto *et al*. (*16*) evaluated the probiotic viability and technological characteristics of spray-dried juçara pulp fermented by *Lactobacillus reuteri* LR92 using maltodextrin, gum arabic and gelatin as the carrier agents. To our knowledge, this is the first study to assess the influence of the processing conditions on the anti- and prooxidant properties of fermented juçara pulp.

The objective of this study is to evaluate *in vitro* the anti- and prooxidant effects of spray-drying and room temperature storage on juçara pulp fermented with *L. reuteri* and *L. plantarum,* using gum arabic, maltodextrin and gelatin as the carrier agents.

## MATERIALS AND METHODS

### Materials

Juçara fruits were harvested from *Euterpe edulis* palm trees grown on the Bimini Farm (Rolândia, Paraná, Brazil). *Lactobacillus reuteri* LR92 (DSM 26866) and *Lactobacillus plantarum* BG112 (LMG 23520) were purchased from Clerici-Sacco (Cadorago, Italy), maltodextrin 10 DE was purchased from Ingredion (Westchester, IL, USA), gum arabic from Nexira SAS (São Paulo, Brazil), and bovine gelatin 220 Bloom from PB Leiner (Acorizal, Mato Grosso, Brazil). Glycerol, sodium hydroxide, ethanol, acetate buffer, DPPH solution, Trolox, luminol and phorbol myristate acetate (PMA) were all obtained from Sigma-Aldrich, Merck (São Paulo, Brazil).

### Fermentation of juçara pulp with L. reuteri and L. plantarum

Juçara fruit pulp was prepared as by Guergoletto *et al*. (*17*). In short, the fruits were sanitized for 30 min in water and chlorine (200 mg/kg), then the grains were rinsed and pulped with clean water (1:1) (fruit/water) using a pulper (Macanuda DM-Ji-05; Joinville, Brazil). The pulp was pasteurized in a water bath at 80 °C for 1 min and frozen at -20 °C until further use.

The bacterial strains (0.01% *m/V*) were frozen in pasteurized juçara pulp containing 20% (*V/V*) sterile glycerol. Prior to the assay, inoculum was activated in the juçara pulp and incubated at 37 °C for 18 h. The juçara pulp was fermented for 30 h at pH=5.7 and 5.0 (adjusted with 0.1 M NaOH) and 32 °C and 25 °C for *L. reuteri* and *L. plantarum*, respectively, as described by Guergoletto *et al*. (*18*). After fermentation, the phenolic content and antioxidant capacity were determined according to the methods described in *Analytical procedures*.

### Spray-drying of fermented juçara pulp using different carriers

Three drying agents (gum arabic, maltodextrin and gelatin) were evaluated separately as carriers for the spray-drying process. Each was added to the fermented juçara pulp (with *L. reuteri* or *L. plantarum*) under aseptic conditions at 10% (*m/V*) (*19*), obtaining six samples. The samples containing maltodextrin or gum arabic were stirred for 10 min at room temperature, and the samples with gelatin were stirred for 20 min at 45 °C (*16*). Samples were spray-dried with a laboratory-scale spray dryer (LabPlant SD-05; Huddersfield, UK) equipped with a 0.7-mm nozzle and the main chamber (500 mm×215 mm). Prior to drying, the dryer conditions were uniformly maintained for 10 min with distilled water. The six samples (two bacteria strains×three drying agents) were continually stirred with a magnetic stir bar and fed into the main chamber *via* a peristaltic pump with a drying air flow of 60 m^3^/h and compressor air pressure of 0.11 MPa. The feed flow rate was 0.52 L/h, and the inlet temperature was (150±1) °C. Low input temperature conditions were selected for higher bacterial survival during drying, as described by Mestry *et al*. (*20*) and Anekella and Orsat (*21*), and the cellular viability results by Guergoletto *et al*. (*16*). Total phenolics, antioxidant capacity measured by DPPH^•^ radical scavenging assay, and oxidative stress in the neutrophil burst were measured before and after spray-drying.

The juçara pulp fermented by *L. reuteri* was chosen for storage analyses based on the neutrophil burst results. For these analyses, the samples were packed in biaxially oriented polypropylene (BOPP), with a light barrier and under vacuum and stored at 25 °C (representing ambient temperature) for 90 days. Every 30 days, total phenolic content, DPPH^•^, and prooxidant effects from the generation of free radicals was measured (*i.e.* measurements on day 0, 30, 60 and 90). These parameters were chosen because the powdered products are usually stored at room temperature for longer than one month (*22*).

### Analytical procedures

#### Total phenolic compounds and DPPH^•^ free radical scavenging assay

Extracts of the fermented and spray-dried juçara pulp samples were obtained with 80% ethanol (at a ratio 1:10 (*m*/*V*)), agitation at 200 rpm (orbital shaker MA 140/CFT; Marconi, São Paulo, Brazil) for 20 min, and centrifugation at 2000×*g* (centrifuge 5804 R; Eppendorf, Hamburg, Germany) for 10 min. The extracts were stored at -22 °C. The total content of phenolic compounds was determined using the Folin-Ciocalteu method (*23*) and a gallic acid standard solution *c*=0.1 to 0.5 mM. The results were expressed in mg gallic acid equivalents (GAE) per g sample, on dry mass basis.

To measure the antioxidant capacities of the fermented and spray-dried juçara pulp extracts, we used the DPPH^•^ assay of Brand-Williams *et al*. (*24*), with some modifications. Aliquots of each extract (0.05 mL) were added to a mixture of 1 mL of 100 mM acetate buffer (pH=5.5), 1 mL absolute ethanol, and 0.5 mL DPPH^•^ solution (250 μM). After 30 min of reaction in darkness at 25 °C, the absorbance was measured at 517 nm with a spectrophotometer (Libra S22; Biochrom, Cambridge, UK). The antioxidant capacity was measured using the standard Trolox curve in the range from 0.5 to 20 μM, and the results were expressed in μM Trolox equivalents per g sample, on dry mass basis.

#### Reactive oxygen species production by the neutrophils (respiratory burst)

ROS production by the neutrophils was evaluated by chemiluminescence in a multilabel plate reader (VICTOR X-3; PerkinElmer, Billerica, MA, USA), according to an adapted method of Freitas *et al*. (*25*) and Huber *et al*. (*4*). First, fermented juçara pulp was freeze-dried, and then, spray-dried samples were diluted in water to *γ*=20 μg/mL. Human neutrophils used as a reagent were isolated from whole blood of one healthy young female volunteer, through gradient density centrifugation, and the neutrophil burst was induced by PMA in the presence of fermented juçara pulp samples or phosphate-buffered saline (PBS; control group), according to de Farias *et al.* (*26*). Blood used for this technique was taken according to the protocol no. 222/2011 (*27*), approved by the Ethics Committee on Research Involving Human Subjects of the State University of Londrina, Brazil. The reaction medium in each well consisted of 200 µL neutrophils (2.5·10^6^ cells/mL), 50 µL luminol (20 mM), 10 μL diluted juçara pulp extracts (20 μg/mL) and 50 µL PMA (5 mM). After fast homogenization, absorbance readings (response range *λ*=300-620 nm) were conducted for 60 min (one read per min) at (30±1) °C. Each experimental group had at least 14 replicates and the results were expressed as count per minute (cpm). For the statistical analysis, we used the peak value of each curve, independent of the time when it occurred (*26*).

#### Measurement of free radical generation by electronic paramagnetic resonance

Free radical generation was determined in the juçara pulp fermented by *L. reuteri* on days 0 and 90 of storage at 25 °C. Samples of the unfermented and lyophilized juçara pulp, maltodextrin, gum arabic, gelatin and lyophilized bacteria were also analyzed to understand the origins of the free radicals. The measurements were conducted by electronic paramagnetic resonance (EPR) on a JEOL spectrometer (JES-PE-3X; Tokyo, Japan) operating on X-band (approx. 9.5 GHz) and a magnetic field modulation at 100 kHz. The measurements were performed with powder samples using 3-mm quartz tubes and the magnetic field marker MgO:Mn^2+^, which was kept in the cavity of the EPR equipment. The spectra were recorded, and the results were expressed as the number of free radicals (species) generated per g sample.

### Statistical analysis

The total phenolic content and the antioxidant capacity determined by the DPPH^•^ method were expressed as mean value±standard deviation (S.D.) and compared using a Tukey’s *post hoc* test with a 95% confidence level in software Statistica v. 7.0 (*28*). The results were considered statistically significant if p<0.05. The results of the respiratory burst were first analyzed using a Gaussian distribution (Shapiro-Wilk test) and homogeneity of variance (Levene’s test). In the absence of a normal distribution and homogeneity of variance, the data were analyzed *via* a non-parametric Kruskal-Wallis test, complemented with Dunn’s test. The statistical analyses were conducted in GraphPadPrism^®^ software v. 3.02 (*29*).

## RESULTS AND DISCUSSION

### Effects of fermentation and spray-drying on the antioxidant potential and inhibition of ROS in juçara pulp

Table 1 shows the results of the total phenolic content and DPPH^•^ radical scavenging analyses. Fermentation (with both bacterial strains) increased the total phenolic content and scavenging of free DPPH radicals in the juçara pulp (p<0.05). This is consistent with the results of our previous study (*18*). However, spray-drying caused a significant decrease in the observed values in both assays. Total phenolic content of fermented juçara pulp spray-dried with gum arabic, maltodextrin and gelatin was 65, 78 and 85% lower than the fermented and non-dried samples, respectively. Interestingly, total phenolic content of the juçara pulp fermented with either bacterial strain and spray-dried with gum arabic was higher than those spray-dried with maltodextrin or gelatin.

**Table 1:** Total phenolic content and antioxidant capacity on dry mass basis of juçara pulp fermented by *Lactobacillus reuteri* and *L. plantarum* and spray-dried with different carrier agents

Sample	*w*(total phenolics as GAE)/(mg/g)		Antioxidant capacity (DPPH^•^) as TE/(μM/g)	
*L. reuteri*	*L. plantarum*	*L. reuteri*	*L. plantarum*
Unfermented pulp	(36.44±0.06)^bA^	(35.40±0.02)^bA^	(1054±5)^bA^	(1049±11)^bA^
Fermented pulp	(43.9±0.1)^aA^	(41.3±0.1)^aA^	(1366±14)^aA^	(1317±19)^aA^
Spray-dried with: Gum arabic	(15.2±0.3)^cA^	(14.0±0.3)^cB^	(260±24)^cA^	(172±10)^cB^
Maltodextrin	(9.7±0.1)^dA^	(9.2±0.2)^dA^	(144±22)^dA^	(132±10)^dB^
Gelatin	(6.8±0.3)^eB^	(7.7±0.2)^eA^	(118±16)^eA^	(119±17)^dA^

Results are expressed as mean value±S.D. (*N*=3). Different superscripted lowercase letters in the same column denote significant differences from a Tukey’s *post hoc* test (p<0.05). Different superscripted capital letters in the same row for the same analysis denote significant differences according to the Tukey’s *post hoc* test (p<0.05). GAE=gallic acid equivalent, TE=Trolox equivalent

The reductions of fermented juçara pulp spray-dried with gum arabic, maltodextrin and gelatin were also significant for the DPPH^•^ method (Table 1); however, there were bacterial strain-dependent effects. The activities of *L. reuteri* fermented pulp dried with gum arabic and maltodextrin were 34 and 8% higher, respectively, than the samples fermented with *L. plantarum* and dried with the same carriers. There were no significant differences in spray-drying with gelatin between the bacterial strains.

**Fig. 1:**
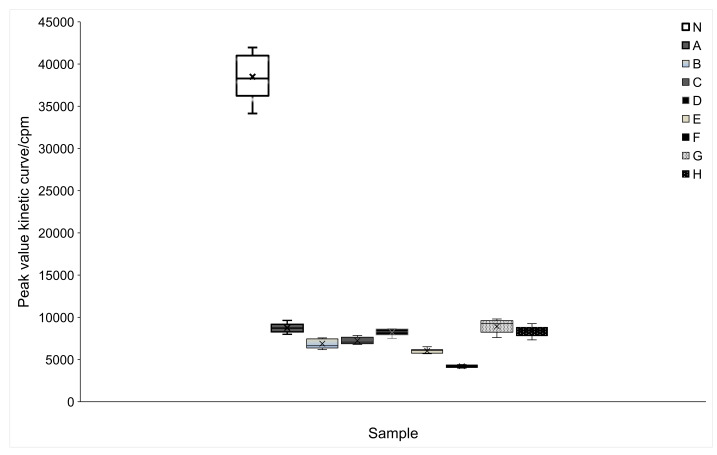
The activation of human neutrophils by phorbol myristate acetate (PMA) in the presence of fermented juçara pulp extracts and those spray-dried with different carrier agents (20 μg/mL). The results were analyzed by Kruskal-Wallis, supplemented with Dunn's test (p<0.001) and compared to the control. For each replicate, the peak value was determined from the kinetic curve and expressed in counts per minute (cpm). N=control, A=juçara pulp fermented with *Lactobacillus plantarum*, B=juçara pulp fermented with *L. reuteri*, C=dry juçara pulp fermented with *L. plantarum* and spray-dried with gum arabic, D=dry juçara pulp fermented with *L. reuteri* and spray-dried with gum arabic, E=dry juçara pulp fermented with *L. plantarum* and spray-dried with gelatin, F=dry juçara pulp fermented with *L. reuteri* and spray-dried with gelatin, G=dry juçara pulp fermented with *L. plantarum* and spray-dried with maltodextrin, and H=dry juçara pulp fermented with *L. reuteri* and spray-dried with maltodextrin

The reactive oxygen species (ROS) released during the reaction can conveniently be detected by the chemiluminescent reagent luminol, and after every minute, the equipment detects these species. When samples are added to phorbol myristate acetate (PMA) and neutrophil cells, it is possible to detect a reduction of the absorbance readings due to the production of fewer species in the presence of antioxidant compounds. When the neutrophils were stimulated with PMA in the presence of the all tested samples, there was a significant inhibitory effect on ROS production (p<0.001), as demonstrated by the significantly lower peak values of the samples than of the control (Fig. 1). Among the samples, the ones fermented with *L. reuteri* and spray-dried with gum arabic were more effective in inhibiting ROS produced by the neutrophils (0-10 000 cpm). These results indicate that the fermented pulp and the spray-dried pulp had an antioxidant effect *in vitro*. This effect occurred through the elimination of the dioxide anion (O_2_ˉ) in the respiratory burst of the neutrophils via electron donation, measured by DPPH^•^ method.

The antioxidant capacity of the juçara pulp may be associated with the presence of phenolic compounds and anthocyanins, whose chemical structures provide reducing power (*7*, *26*). The bacterium-specific fermentation differences observed in this study may stem from the characteristics of each species. According to Kullisaar *et al*. (*30*), some species of lactic acid bacteria possess antioxidant enzymes such as superoxide dismutase (SOD), NADH oxidase, NADH peroxidase and catalase, and *L. reuteri* has a greater ability to reduce oxidative damage during endotoxic shocks than other *Lactobacillus* species (*31*).

The bioactive compounds of plants can degrade during spray-drying, but in this study, the fermented and spray-dried juçara pulp inhibited the neutrophil oxidative burst better than the pulp extracts. This result can be explained by the potential protective effects of the carriers (gum arabic, maltodextrin and gelatin) on the antioxidant activity and bacterial cells. Thus, our results suggest that the spray-drying does not affect the inhibitory effects on ROS production.

The fermented pulp and that dried with gum arabic had higher contents of phenolic compounds and greater protective effects on the antioxidant components of the fermented juçara pulp powder. Oliveira *et al.* (*32*) found that the powders produced from these materials contain high bulk density particles with less air, which may lower the rate of oxygen diffusion and, consequently, decrease the oxidation of the compounds. Tonon *et al*. (*22*) evaluated the use of carrier agents (maltodextrin, gum arabic and tapioca starch) in the drying of açaí pulp and found no differences in the anthocyanin content and antioxidant capacity when using maltodextrin or gum arabic.

### Evaluation of the total phenolic compounds, antioxidant activity, and free radical generation of stored juçara pulp fermented with L. reuteri and spray-dried with different carriers

Juçara pulp was fermented with *L. reuteri*, spray-dried, and then stored at 25 °C for 90 days. The samples were evaluated every 30 days, due to their high antioxidant capacity observed in the previous experiment. The total phenolic content and antioxidant capacity (measured by the DPPH^•^ method) of the samples decreased significantly over the 90 days (p<0.05; Table 2). These changes were less pronounced when the samples were dried with maltodextrin (24% decrease of total phenolic content and 19% of DPPH scavenging), followed by gum arabic (decrease of 50.7 and 69%) and gelatin (decrease of 61 and 78%). Tonon *et al*. (*22*) observed higher anthocyanin retention and higher antioxidant activity during storage with maltodextrin 10 DE. According to the authors, higher stability is associated with lower water activity, which is consistent with our previous study of fermented juçara pulp spray-dried with maltodextrin, gum arabic and gelatin (*16*). Rodríguez-Hernandez *et al*. (*33*) attributed higher stability to the better binding properties of maltodextrin 10 DE, which has a higher polymerization degree.

**Table 2:** Total phenolic content and antioxidant capacity on dry mass basis of stored juçara pulp fermented with *Lactobacillus reuteri* and spray-dried with different carrier agents

*t*/day	*w*(total phenolics as GAE)/(mg/g)			Antioxidant capacity (DPPH^•^) as TE/(µM/g)		
Gum arabic	Maltodextrin	Gelatin	Gum arabic	Maltodextrin	Gelatin
0	(14.0±0.3)^a^	(9.8±0.2)^a^	(5.5±0.1)^a^	(230±24)^a^	(121±14)^a^	(120±24)^a^
30	(10.1±0.2)^b^	(9.71±0.09)^a^	(2.97±0.08)^b^	(120±8)^b^	(107±10)^b^	(58±13)^b^
60	(7.7±0.3)^c^	(9.1±0.1)^b^	(1.96±0.07)^c^	(97±5)^c^	(103±9)^b^	(28±2)^c^
90	(6.9±0.2)^d^	(7.9±0.2)^c^	(1.70±0.06)^c^	(89±5)^c^	(92±7)^c^	(26±13)^c^

Results are expressed as mean value±S.D. (*N*=3). Different superscripted lowercase letters in the same column denote significant differences according to the Tukey’s *post hoc* test (p<0.05).

GAE=Gallic acid equivalent, TE= Trolox equivalent

**Fig. 2:**
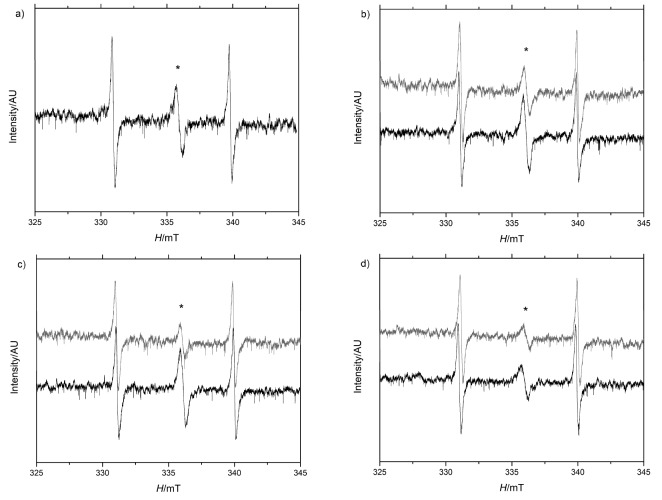
Electronic paramagnetic resonance spectra of juçara pulp fermented with *Lactobacillus reuteri* on day 0 (gray lines) and 90 (black lines) of storage at 25 °C using different carrier agents: a) lyophilized juçara extract (control), b) gum arabic, c) maltodextrin, and d) gelatin. *indicates free radical resonance. Side signals represent the magnetic field marker MgO:Mn^2+^

In the electronic paramagnetic resonance (EPR) analysis, a small free radical signal (Fig. 2) was detected in all of the samples and the unfermented and lyophilized juçara pulp. Maltodextrin, gum arabic, gelatin and the lyophilized bacteria were also analyzed to clarify the origins of the free radicals; however, none presented EPR signals. The EPR analysis of free radicals in the dried and stored pulp revealed similarities across the samples, with an increase in the number of species per g sample after 90 days of storage (Table 3 and Fig. 2).

**Table 3:** Electronic paramagnetic resonance parameters of lyophilized juçara pulp fermented by *Lactobacillus reuteri* and spray-dried with different carrier agents

Sample	*t*(storage)/day	*g*	Δ*H*_pp_/mT	*N*(free radical species)/g
Lyophilized juçara pulp	0	(2.005±0.005)	(0.496±0.005)	(3.034±0.000)·10^15^
Spray-dried with: Gum arabic	0	(2.004±0.005)	(0.405±0.005)	(0.781±0.005)·10^15^
Maltodextrin	0	(2.004±0.005)	(0.400±0.005)	(0.784±0.005)·10^15^
Gelatin	0	(2.004±0.005)	(0.445±0.005)	(0.741±0.005)·10^15^
Gum arabic	90	(2.004±0.005)	(0.469±0.005)	(3.650±0.005)·10^15^
Maltodextrin	90	(2.004±0.005)	(0.423±0.005)	(2.674±0.005)·10^15^
Gelatin	90	(2.004±0.005)	(0.531±0.005)	(2.642±0.005)·10^15^

*g*=spectroscopy factor, Δ*H*_pp_=peak-to-peak line width of the derivative of the resonance signal

High-temperature dehydration may degrade anthocyanins (*34*) and oxidize some lipid compounds, leading to the formation of or increase in the free radicals in dehydrated samples (*35*). However, in this study, the characteristics of the spectroscope factor (*g*) and the peak-to-peak line width (Δ*H*_pp_) of the EPR spectra were similar for both the fermented and spray-dried juçara pulp and the lyophilized (control) unfermented pulp, suggesting that the atomization process did not influence the formation of free radicals, and that its origin can be attributed to the juçara itself or to the method of extraction of its pulp. In addition, the antioxidants present in juçara pulp might have acted to prevent the formation of more free radicals or the formation of a less active radical (*36*), because phenolic compounds are present in plants as a form of protection against ultraviolet and visible light (*37*). Similar values ​​of the Δ*H*_pp_ factor suggest as a strong indicator that the same radical was present in all the juçara pulp samples and the low *g*-factor values ​​are characteristic of unpaired electrons allocated to oxygen and carbon present in the pulp of juçara with a low connection to the spin orbit (*38*).

The weak singlet signal obtained (*g*=2.004) and peak-to-peak line width (Δ*H*_pp_=0.4000±0.0005 mT) are common in plants and result from their metabolic activities (*39*). Although their origin is not fully understood, it has been suggested that these compounds are semiquinone or lignin-like free radicals, remnants of plant metabolism or produced by the oxidation of polyphenols or plant fatty acids (*40*). Studies suggest that the lignin is degraded by extracellular enzymes in smaller portions, which makes it susceptible to absorption by microbial cells, where it is then converted into phenols and quinones that are released with oxidative enzymes into the environment to be polymerized by the mechanisms of free radical formation (*41*-*43*). Alternatively, the process of pulping açaí and juçara in a depulper (or mixer) with water introduces excess air and may encourage the oxidation of fruit lipids. Further study is needed to elucidate the origins of free radical formation in juçara pulp.

Spray-drying requires high temperatures and is widely used for the dehydration of food and pharmaceutical products, due to its short time requirements and applicability to products with sensitive components (*11*). Our results suggest that spray-drying of fermented juçara pulp does not influence the production or proliferation of free radicals. We observed similar free radical content across juçara samples and the increase in the number of species after 90 days of storage was still lower than the content of radicals observed in herbal studies (10^18^ spin/g) (*9*) and sterilized mulberry leaves (10^18^ spin/g) (*38*). The increase in free radicals may be related to reduced antioxidant capacity and phenolic contents after storage. Katsube *et al*. (*34*) reported that the anthocyanins present in juçara are very sensitive to storage, particularly the temperature and contact with oxygen, which can directly influence the antioxidant capacity of the fermented and dehydrated juçara pulp. Even though the fermented and dehydrated juçara pulp showed reduced antioxidant capacity and a higher number of free radical species after storage, it is still noteworthy that both processes were effective in maintaining the beneficial properties, as evidenced by the lack of an increase in the intensity and the types of free radicals (as observed by EPR soon after drying). This result is supported by the protective effects observed in the neutrophil burst assay.

## CONCLUSION

The fermentation of juçara pulp with *Lactobacillus reuteri* LR92 (DSM 26866) and *Lactobacillus plantarum* increased the antioxidant capacity and total phenolic content, whereas spray-drying decreased these values. However, drying did not influence the ability to scavenge reactive oxygen species in the neutrophil respiratory burst, and the samples spray-dried with gum arabic were more resistant to oxidative damage. Unfermented juçara pulp naturally contained free radicals, as detected by electronic paramagnetic resonance (EPR), but the radicals remained stable after drying. This may have contributed to the retaining of beneficial effects, as indicated in the neutrophil test. Prolonged storage at room temperature was detrimental to the oxidative stability of the products, as evidenced by higher numbers of free radicals and lower values of the total phenolic content and antioxidant capacity.
